# The roles of RFamide-related peptides (RFRPs), mammalian gonadotropin-inhibitory hormone (GnIH) orthologues in female reproduction

**DOI:** 10.22038/IJBMS.2018.30520.7355

**Published:** 2018-12

**Authors:** Huimei Wang, Arezoo Khoradmehr, Mohammad Jalali, Mohammad Saied Salehi, Kazuyoshi Tsutsui, Mohammad Reza Jafarzadeh Shirazi, Amin Tamadon

**Affiliations:** 1 Department of Integrative Medicine and Neurobiology, School of Basic Medical Sciences; Institute of Acupuncture and Moxibustion, Fudan Institutes of Integrative Medicine, Fudan University, Shanghai, China; 2 Research and Clinical Center for Infertility, Yazd Reproduction Sciences Institute, Shahid Sadoughi University of Medical Sciences, Yazd, Iran; 3 The Persian Gulf Marine Biotechnology Research Center, The Persian Gulf Biomedical Sciences Research Institute, Bushehr University of Medical Sciences, Bushehr, Iran; 4 Department of Physiology, Faculty of Biological Sciences and Technology, Shahid Beheshti University, Tehran, Iran; 5 Laboratory of Integrative Brain Sciences, Department of Biology and Center for Medical Life Science, Waseda University, Tokyo, Japan; 6 Department of Animal Science, College of Agriculture, Shiraz University, Shiraz, Iran

**Keywords:** Female, Gonadotropin-inhibitory- hormone, Mammals, RFamide-related peptide Reproduction

## Abstract

**Objective(s):**

To benefit from reproduction and deal with challenges in the environmental conditions, animals must adapt internal physiology to maximize the reproduction rate. Maladaptive variations in the neurochemical systems and reproductive system can lead to manifestation of several significant mammalian reprocesses, including mammalian ovarian lifespan. RFamide-related peptide (RFRP, Rfrp), mammalian orthologues of gonadotropin-inhibitory hormone (GnIH), which is a regulator to prevent the gonadotropin-releasing hormone (GnRH) neural activity, is known to be related to reproductive traits. This review aimed to summarize recent five-year observations to outline historic insights and novel perspectives into the functions of RFRPs in coding the mammalian reproductive physiology, especially highlight recent advances in the impact on RFRPs in regulating mammalian ovary lifespan.

**Materials and Methods:**

We reviewed the recent five-year important findings of RFRP system involved in mammalian ovary development. Data for this review were collected from Google Scholar and PubMed using the RFRP keyword combined with the keywords related to physiological or pathological reproductive functions.

**Results:**

Recent discoveries are focused on three major fronts in research on RFRP role in female reproduction including reproductive functions, energy balance, and stress regulation. The roles of RFRPs in various development phases of mammal reproduction including prepuberty, puberty, estrous cycle, pregnancy, milking, menopause, and/or ovarian diseases have been shown.

**Conclusion:**

Overall, these recent advances demonstrate that RFRPs serve as critical mediators in mammalian ovarian development.

## Introduction

For any species or individual to survive and thrive, there is a necessary biological need for the reproductive system to develop structures and mechanisms in order to ensure the greatest fertility. Physiological and psychological mechanisms facilitating the process of reproduction must be selected through evolution. The reproductive complexity has been reported on biological mechanisms of gonadotropin-releasing hormone (GnRH) isolated from both non-mammals and mammals and considered as the sole regulator of the hypothalamus-pituitary-gonad (HPG) axis for a long time. However, since the seminal discovery made by Tsutsui *et al*. (1), a gonadotropin-inhibitory hormone (GnIH) in the brain of quail producing inhibitory regulations on the reproductive axis, three essential questions have arisen: What brain sites are involved in the innervation of GnIH neurons? What role does GnIH play in ovarian development? What factors affect GnIH? 

**Figure 1:**
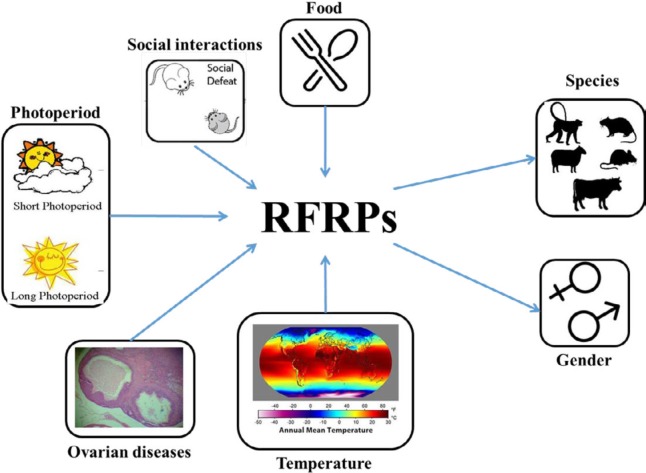
Relationships between different factors and RFamide-related peptides (RFRPs), mammalian gonadotropin-inhibitory hormone (GnIH) orthologues. Food, social interactions, photoperiod, and ovarian diseases are the common factors underlying the effects of RFRPs of mammalian ovarian development. For each of these mediators, different mammals and genders were studied. We suggest using various behavioral paradigms and intervention means to assess the influence of RFRPs on ovarian development periods, especially among mammals with different genetic backgrounds

In the recent decades, enormous advances have accumulated in elucidating the physiological mechanisms of GnIH underlying reproductive processes in animals. Mammalian GnIH orthologues, RF (Arg-Phe) amide-related peptides (RFRPs), including RFRP-1 and RFRP-3, are considered as essential regulators of GnRH neurons (2) and HPG axis (3). Moreover, the field has also started to appreciate that various functionalities of RFRPs are affected by various factors, including social interactions (4), photoperiod (5), temperature (6), food (7), and some diseases (8) in ovarian developmental periods. Additionally, RFRPs play different roles in different genders (5) and species (9, 10) in the reproductive development (Figure 1). Furthermore, the discovery of RFRPs’ functionality from prepuberty to menopause created novel perspectives on how different ovarian developmental periods might be responsive to regulate reproduction through RFRPs.

**Figure 2:**
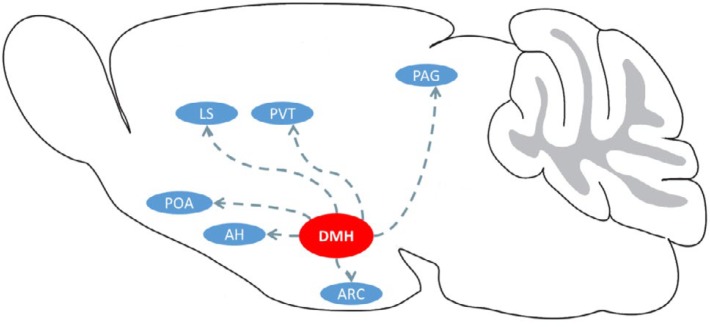
A simplified schematic summarizing the potential neural pathways of RFRP neurons in the hypothalamus in mammals. Abbreviations: DMH, Dorsomedial hypothalamic nucleus; POA, preoptic area; LS, lateral septum; PVT, paraventricular nucleus of the thalamus; PAG, periaqueductal gray; ARC, arcuate nucleus

In the recent five years, evidence began to mount that RFRPs have significant repressive influence on mammalian reproduction regulation in various species including rat (11, 12), mouse (13), desert jerboa (14), hamster (10, 15, 16), pig (17, 18), sheep (19), goat (20), cow (21), monkey (22), and human (23). Moreover, localization and projections of RFRP propeptides vary in different mammalian species (Table 1, Figure 2).

In a compelling set of experiments on female hamsters, it has been demonstrated that the RFRP system exerts variable effects according to the season, the stage of the estrous cycle, and the time of the day, which are essential for synchronization of different reproductive activity (10). However, recent studies have also provided new insights that RFRP-3 may have no direct effect on controlling the secretion of luteinizing hormone (LH) in the ewe (9). Consistent with this hypothesis, RFRP-3 and GnRH immunoreactivities were unchanged in the bovine hypothalamus both in proestrus and diestrus (21). Therefore, we should not rule out the possibility that some functions mediated by the RFRP system may be different in a variety of species. The RFRP system robustly regulated by photoperiod in male and female Syrian hamsters showed more significant effects on females (5). However, further work is needed to classify the influence of RFRP in different genders and species. 

**Figure 3:**
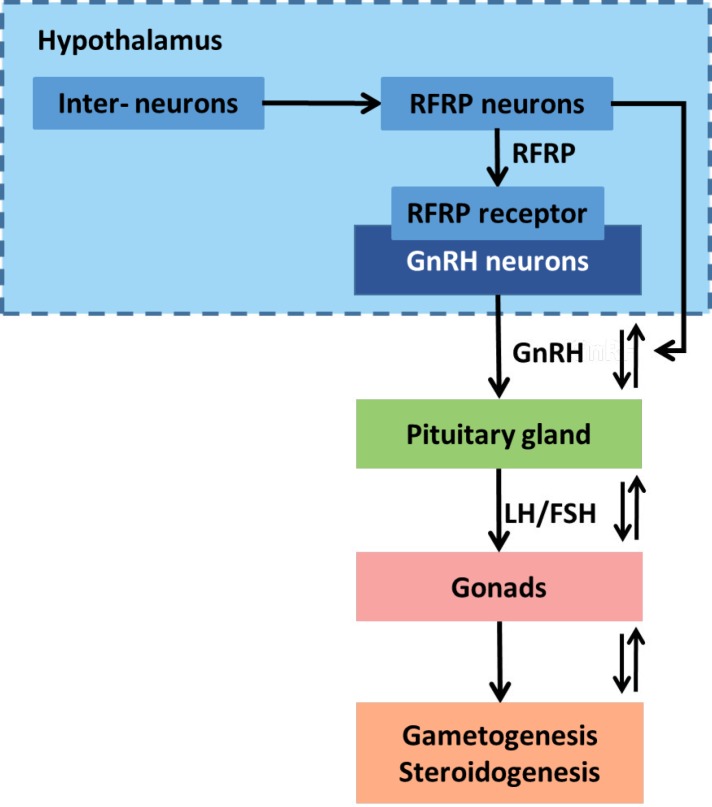
Efficacy of RFamide-related peptide (RFRP) signaling in regulating the reproduction of mammals. RFRP neurons generate an inhibitory activity in the hypothalamus-pituitary-gonad axis (HPG axis) via their G protein-coupled receptor. Abbreviations: GnRH, gonadotropin-releasing hormone; LH, luteinizing hormone; FSH, follicle-stimulating hormone

Here, we review some important findings of the RFRP system involved in the development of mammalian ovary, emphasizing the recent five-year advances. Data for this review were collected from Google Scholar and PubMed using RF-amide-related peptides or RFRP combined with biochemistry, molecular physiology, ovary, prepuberty, puberty, estrous cycle, pregnancy, milking, menopause, and/or ovarian diseases as keywords. Supplemental literature searches used gonadotropin-releasing hormone, GnRH, gonadotropin-inhibitory hormone or GnIH combined with ovary, prepuberty, estrous cycle, pregnancy, milking, menopause, and ovarian diseases as keywords (Figure 3). A better understanding of the RFRP mechanisms underlying mammalian ovarian life-span may give novel insights into reproduction and therapies development for ovarian diseases.

**Biochemistry and molecular physiology of RFRP**

The precursor of RFRPs is synthesized and processed into two mature neuropeptides, RFRP-3 and RFRP-1, characterized by a conserved C-terminal LPXRFamide (X = L or Q) motif (26). However, the structural differences between the two peptides exist in their C terminus by an LPLRFamide for RFRP-1 and LPQRFamide for RFRP-3, and N terminus is evidently different between two peptides among species (27). It has been implicated that RFRP-1 can stimulate prolactin secretion, although it does not have a similar effect on the other pituitary hormones including LH, follicle-stimulating hormone (FSH), adrenocorticotropin, growth hormone, and thyroid-stimulating hormone, suggesting that RFRP-1 may be involved in the regulatory functions of endocrine instead of coding reproduction. However, the impacts of RFRP-3 in mammals are even more elusive compared with RFRP-1 on regulating gonadotropins’ secretion (10, 13, 28-30). In addition, these studies were especially focused on the modulation of mammalian reproduction, including activating LH secretion and synthesis (10, 13) and modulating the process of puberty onset (28-30). In this regard, the exact mechanisms of RFRP-3 involved in mammalian ovarian development still require further confirmation. 

**RFRP in mammalian ovary life-span**

Ovarian life-span considerably influences the reproductive process. Although apparent differences exist in the sequence and timing of ovarian development between mammalian species, ovaries of different mammals largely develop in a similar way. It is now clearly understood that the ovary, a multi-compartmental gonad in females, causes differentiation and release of the mature eggs or oocytes, produces steroids stimulating the development of female secondary sexual properties and supports functionalities of pregnancy. Moreover, the ovarian hormones also play an essential role in the processes from puberty development to ovulation and reproductive cycle (31). Animal and human studies have begun to recognize GnRH and RFRP are activated during mammalian reproduction. GnRH was once considered the single driver of gonadotropins and secretion of gonadotropins in the modulation of reproduction in mammals. Recently, extensive knowledge has accumulated on the investigation of reproductive mechanisms of RFRP, which is considered a potential inhibitory regulator of GnRH release in mammals. They indicated that the activity and expression of RFRP could modulate gonadotropin and GnRH secretion and are strongly linked to infertility and sexual behavior (32). 

Extensive knowledge is accumulating on the neuroanatomical interactions that the gonadal GnIH system, independently from the brain, directly responds to changes in circulating glucocorticoids (46). In *in vivo* and *in vitro* conditions, RFRP-3 suppresses the secretion of gonadotropins in female sheep and rats (47, 48). Several studies also showed that RFRP-3 neurons project to GnRH neurons and inhibit LH secretion (49-51). Thus, RFRPs hinder the secretion and synthesis of gonadotropin via indirect and direct pituitary actions. In female sheep, it was proposed that RFRP-3 inhibited the pulse amplitude of LH and decreased gonadotropin secretion stimulated by GnRH (47). The possibility that the RFRP system plays an essential role in modulating the ovulation and LH surge in female hamsters has been suggested (52). Expanding on this, RFRP-3 inhibits the firing rate of GnRH in mice (53). RFRP-3 might exert its effects on reproduction either indirectly through upstream regulators of GnRH, and/or directly via a subset of GnRH neurons (41). RFRP decreased the expression of GnRH mRNA in a hypothalamic Rfrp-expressing cell model (54).

Together with recordings in avian species (55, 56), RFRP-3 is demonstrated to act as a regulator of HPG axis to support the general reproductive actions, suggesting that RPRPs may play a highly conserved local regulatory role in peripheral reproductive tissues and gonads. However, a large body of recordings suggests that the special local functions of RFRPs can differ across species (Table 2). Below, we discuss the roles of RFRPs in various development phases of mammals’ reproduction.

*Role of RFRP in puberty*

Puberty onset modulated by reproductive neuroendocrine systems, is a complex and well-organized process. It is currently revealed that puberty is regulated via high-frequency hypothalamic GnRH pulsing. Furthermore, GnRH release secretes FSH and LH. Given the classical stimulatory role of GnRH in the regulation of the pituitary-gonadal axis, RFRP-3 is also involved in preventing reproduction as an inhibitory neuropeptide (57). As RFRP signaling exerts its impacts on the reproductive axis through gonadal and pituitary gonadotropins, finding the association of neural projections of RFRP from the hypothalamic nucleus with GnRH provided new possibilities of how RFRP systems may respond to prepubertal and pubertal periods.

Recent studies using common marmoset monkeys have proved a significant surge in the RFRP transcription level in the prepuberty of juvenile monkeys (22). In the early prepubertal period, a strong reduction was observed in the dorsomedial hypothalamic region (DMH) in RFRF-3/c-fos co-expression and RFRP-3 expression (29). This marked decrease in inhibiting RFRP-3 population might demonstrate that inhibition of GnRH neurons diminishes during early puberty in humans (29). Intracerebroventricular injection of RFRP-3 markedly delayed puberty onset of female rats, showing the possibility that RFRP-3 may be correlated with prepubertal increase in growth hormone secretion (11). Similarly, in female mice, the delayed pubertal onset could be due to the suppression of GnRH gene expression under dexamethasone treatment, accounting for the inhibitory signals from GnIH to the preoptic area to some degree (58). To explore the neuroendocrine mechanisms of RFRP-3 in social reproductive inhibition of puberty, it was found that exogenous RFRP-3 inhibits mating behavior and gonadal steroidogenesis in naked mole rats underlying the given inhibited circumstances of puberty (12). Importantly, the role of RFRP-3 in preserving immature reproduction provides proof that stimulatory peptides were not the sole regulators of puberty (59). Neonatal exposure to bisphenol A, which is well understood to lead to advances in puberty, persistent estrus, irregular estrous cycles, and compromised fertility, and can also induce premature puberty in female mammals (59-61). This is proved to result from decreased inhibition of GnRH neurons (59).

GPR47 as the RFRP cognate receptor is expressed in the testes, pituitary, and hypothalamus of mammals. Moreover, neuropeptide FF receptor 2 (NPFFR2), a putative receptor of RFRPs, was revealed to play a crucial role in pubertal onset in gilts (17). RFRP-3 might play a role in reproduction either indirectly by upstream modulators of GnRH or by GPR147 in a small proportion of murine GnRH neurons (41). Based on their prior work, a more careful examination of kisspeptin effects (the Kiss1 gene neuropeptide product) or RFRP-3 on modulating reproductive status revealed that RFRP-3 might affect a subset of neurons derived from kisspeptin in mice, especially in the arcuate nucleus (ARC) of mice. However, no evidence in this study proved that kisspeptin directly modulates reciprocal actions on RFRP-3 neurons (43). Likewise, in humans, RFRP-3/GPR147 may have secondary and modulatory effects on regulation of puberty (29). 

RFRP-3 could suppress the release of pituitary LH effectively through inhibition of transcription and translation of GnRH in female mice, being connected with ovarian development periods and the estrogen signaling pathway (28). Moreover, central administration of RFRP-3 was shown to inhibit LH secretion in female mice when preovulatory LH surge in naive mouse or LH surge was generated through estradiol in ovariectomized mice (13). However, central administration of RFRP-3 had a low effect on LH secretion in both intact diestrus and low dose estradiol-administered ovariectomized mice (13). Low concentration of estradiol as an ovarian sex steroid might affect the mRNA expression of RFRP at the prepubertal stage (62). Expanding on this, sexual maturation progress was observed in rats treated with chronic testosterone, suggesting that this top-down control might be triggered by a peripheral, instead of a central mechanism (63). Additionally, peripheral RFRP-3 had no effect on intact female micee during diestrus or at preovulatory LH surge (13). This showed the effect of cycle stage-dependence on gonadotropin secretion in female mice. On the other hand, fluctuations of RFRP-3 in reaction to food restriction may modulate the HPG axis in prepubertal ewes (64).

An emerging theme is that endogenous RFRP-3 signaling might not play a necessary role in pubertal development, at least in some species suggesting novel hypotheses of how RFRP-3 may control sexual maturation and development (65). Most recently, an exciting novel wave of results has emerged, supporting that GnIH exerts its effects on proper pubertal development via regulating the hypothalamus-pituitary-thyroid (HPT) and HPG axes interactions (66). 

*Role of RFRP in the estrous cycle*

The estrous cycle is the reproductive cycle found in most mammalian females whereby there are recurring periods starting from sexual maturity and ceasing at death, interrupted by anestrous stage or pregnancies and triggered by reproductive hormones. The RFRP gene of pigs has been cloned and characterized (33). Their work showed the close associations with RFRP gene and reproductive traits in pigs. A prominent view supported by data from immunolocalization in the pig ovary in the estrous cycle was that GnIH immunoreactivities and its receptor-GPR147 were localized mostly in the luteal cells in diestrus and metestrus and in the granulosa and theca cells of antral follicles during estrus and proestrus (67). To specifically clarify the potential locations and mechanisms of RFRP-3 action, the direct role of RFRP-3 in the reproductive axis of female pigs *in vitro* has been identified (34). They revealed that different doses of RFRP-3 inhibited the secretion and synthesis of gonadotropin, steroid hormones, and GnRH, and affected gene expression, including GnRH and Kiss1 and protein expressions such as ERK 1/2, cycling B1, and proliferating cell nuclear antigen. Similarly, RFRP neurons in the DMH are found to have a considerable impact on controlling estrous cyclicity in rats (36). In addition, GnRH and LH can be restrained to maintain pulsatile and low-amplitude release by estrogen negative feedback during the major phase of the ovulatory cycle (reviewed by Williams and Kriegsfeld (68). Based on these molecular and morphological results, the evidence suggests that RFRP may exert critical effects on the estrous cycle. 

A current study showed that coordinated expression of RFRP-3 and Kiss1 mRNAs participated in response to estrous cycle in the hypothalamus in rats (37). The relationship between RFRP-3 and Kiss1 has been extensively explored in Syrian male hamsters (44), mice (41) and cows (21). These studies showed that decisive position of RFRP-3 can exert a stimulatory impact on the reproductive axis, most likely through hypothalamic targets. Although kisspeptin neurons do not have any direct reciprocal roles in RFRP-3 neurons, RFRP-3 acts as a stimulator to modulate a small proportion of neurons derived from kisspeptin in mice (43). Another study has shown that the roles of RFRP-3 are developmental status- and sex-dependent in hamsters (16). 

Epidemiological and experimental findings reflected that circadian disruption plays pronounced negative roles in the female reproductive axis. Both GnRH and RFRP-3 neurons sustain their own circadian cycles to ensure inhibition and stimulation of the reproductive axis are coordinated precisely (25). Early recordings of the circadian system revealed interactions between estradiol-sensitive neural circuits, circadian timing, driving preovulatory LH surge, and GnRH secretion (68-70). A number of models in rodents suggest that RFRP-3 and kisspeptin neuronal populations play a key node for integrating and transducing hormonal and circadian signals to the reproductive axis, thus they control the LH surge, precisely and timely (5, 35, 71, 72). Some of these studies were dependent on circadian neural oscillations such as serotonergic and dopaminergic ones to study the age-dependent change in hypothalamic RFRP-3 expression and its relation with gonadal maturation and functions (57, 73, 74) (Sethi and Chaturvedi, 2015, 2016). They found that administration of L-DOPA and 5-HTP in breeder mice induced expression of RFRP-3 neurons in DMH nuclei and altered the testicular activity, simultaneously. In research exploring the photoperiodic regulation effect of goat RFRP gene during reproductive process, RFRP gene was proposed to be indirectly connected with reproductive seasonality and has undergone a selective pressure in sunshine duration in goats (20). 

A recent study has shown that adult females have markedly more RFRP-1-ir per cell being regulated in the estrous cycle compared with male rats (27). Additionally, GnIH-3 was a secreted neurohormone that could be released into the portal blood to reduce the effects of GnRH through modulating pituitary gonadotropes during the anestrous and luteal follicular periods of the estrous cycle in the reproduction phases in ewes (24). In the dorso/ventromedial hypothalamus of jerboa captured in the spring, the RFRP transcription level was observed to increase compared to autumn (14). 

RFRP-3 is proven to repress the reproductive axis via NPFF1R (75) so that lack of its inhibitory pathway partially hindered suppression of gonadotropin in the NPFF1 receptors of null mice in puberty and fertility under metabolic stress (30). GJ14 is a highly potent and specific NPFF1R antagonist with moderate antagonism at NPFF2R. It was observed that RFRP-3 was a promoter of hypothalamus-pituitary-adrenal (HPA) axis in mice, while infusion of GJ14 reversed the anxiogenic and HPA axis-stimulatory impacts of RFRP-3 (42). Interestingly, the findings presented suggest that not only in female animals, but also in male Syrian hamsters, the expression of RFRP was impacted markedly by reproduction condition and photoperiod, which suggests that RFRP in testis partly leads to reproductive inhibition (76). Female sexual behavior and motivation coordinate with the optimal time of fertility (77). RFRP acts as an activator of female proceptive sexual behavior and motivation, and this role is independent of the downstream alterations in the production of sex steroids such as circulating gonadal steroids and kisspeptin (45). As reflected by a multitude of expounded experiments (78-81), RFRP is more likely to play a temporary role in reproductive suppression in response to different physiological stimuli during the breeding season.

*Role of RFRP in pregnancy*

Pregnancy is a complex period of reproduction during which females carry one or more live offspring from implantation in the uterus through gestation and from egg fertilization to parturition. Plasma progesterone, which is synthesized by the nervous system from cholesterol de novo (82, 83), is a prominent indicator measured by radioimmunoassay through pregnancy and lactation in females (82). It has been clearly shown that treatment with progesterone or treatment with estradiol plus progesterone significantly decreased activation of RFRP-3 cells in the Syrian hamster DMH, whereas treatment with estradiol alone increased activation of RFRP-3 cells in the hamster DMH (84). Partly owing to their neuroanatomical connectivity, GnIH neurons have been proposed to be regulated by progesterone in seasonally breeding birds (85). Furthermore, it has been shown that GnIH adaptively reacts to unpredictable changes in the environment in a potentially conserved way in female periods in starlings and rats, especially in the period of parental care (39). The responsive patterns in RFRP-3 mRNA may derive from joint exposure to suppression of LH levels and increased levels of progesterone in pregnant rats (86). These results provide an anatomical and physiological basis for RFRPs acting as effectors of reproduction function of mammals.

Given that it has been determined that serum LH tends to decrease to the lowest level at mid-pregnancy and recover at the end of pregnancy, it is not surprising that RFRP is associated with secretion of GnRH and LH (87). An emerging discovery is that the levels of GnRH and LH secretion were decreased in the hypothalamus of pregnant rats, promoting the hypothesis that GnRH and LH secretion may be controlled by transcription conditions of RFRP-3 and LH genes (87). Similar to RFRP-3, kisspeptin is also a neuropeptide belonging to the large RFamide peptide subfamily. A recent research has discovered that in mice kisspeptin and RFRP-3 act as strong modulators to stimulate and inhibit GnRH and LH secretion, respectively (43). The expression of hypothalamic *Kiss1* and *Kiss1r* mRNAs was characterized in pregnant and non-pregnant mice, the circadian input to Kiss1 in pregnancy is absent despite normal clock gene expression and high degrees of gestational estradiol (88). Therefore, a plausible explanation consistent with these data is that perturbations of kisspeptin-specific diurnal rhythm may operate in the non-pregnant state. However, further recordings are still needed to report potential roles of RFRP in pregnancy of mammals.

*Role of RFRP in milking*

The effects of milking are associated with regulation of reproductive endocrine hormones regulating from lactation to negative energy balance effects on neuroendocrine pathways. In various mammalian species, ovulation and follicular maturation are hindered in lactation (89). It is well understood that the inhibition of the estrous cycle mostly results from the inhibition of GnRH and LH secretion (90). RFRP serves as the potential regulator of LH and GnRH secretion, and has been assumed in biological mechanisms in the process of lactation. 

Early recordings of the increase of RFRP-3 transcription in DMH may inhibit GnRH release at the stage of milk production increase in rats, compared with no variation in *Kiss1* mRNA expression found in the ARC of the same rats, suggesting the lower activity of ARC on expressing the gene postpartum (91). In particular, one study in the DMH of rats has demonstrated that RFRP mRNA expression is stimulated by the suckling intensity and increased litter size, which may have a strong effect on lactation anestrus (40). Conversely, RFRP-3 genes are shown to increase the levels of the reproductive hormones to induce an immune response but have a minor role in improving twinning rate and litter sizes of ewes (19). A more careful examination of constructing DNA vaccines with the fragment of inhibin-α (INH) using the RFRP-3 gene in mice revealed that p-TPA-SINH/TPA-SFRFP inserted with *INH* and *RFRP* genes is successfully constructed with good immunogenicity, providing the possibility to increase litter size, effectively (19). 

*Role of RFRP in menopause*

Menopause, the point when females end showing the estrous cycle, is connected with endocrine regulation in pituitary, hypothalamus, and gonads. It is now well understood that aging is correlated with HPG axis disruption in the downstream and decreased GnRH release. Although GnRH is the most famous hormone for pulsatile secretion to interact with the pituitary gland, which in turn interacts with the ovaries through LH and FSH release from puberty onset (92), the RFamide peptide superfamily, including 26/43 RFa, kisspeptins, GnIH and RFRPs, has recently attracted considerable attention owing to its role in reproductive aging that may be a factor for regulating menopause (5). 

A recent rat-based study has proven that the expression of RFRP-3 and its receptor locally increase in the ovarian life-span with age (92). In the same study, the transcriptional increase of RFRP-3 is exhibited in the hypothalamus at middle age earlier than changes in estrous cyclicity (38). Furthermore, this increase is proven to be transient and followed by decreases in both the mRNA expression of GnRH and kisspeptin. Likewise, RFRP-3 inhibits the levels of LH secretion in postmenopausal women; however, compared with men administrated with GnIH/RFRP-3, the secretion of LH stimulated by kisspeptin is not decreased (23). 

HPG and the HPT axes are integrated networks that can modulate both androgenization and estrogenization involving embryonic infancy, puberty and adult sexual maturation. Abnormalities in the HPT axis may induce some functional changes of male aging, resulting in sarcopenia, adiposity, osteopenia, insulin resistance, and impaired recovery from prolonged critical illness (93, 94). Further researches should resolve whether the same rules apply to female aging, which is largely correlated with dysregulation of the HPG axis, leading to the climacteric syndrome.

**Role of RFRP in ovarian diseases**

Abnormalities of reproductive hormones and reproductive dysregulation are characterized by symptoms of LH hypersecretion, hyperandrogenism that leads to hirsutism, follicular maturation dysregulation resulting in ovulatory disturbance, and subfertility (95, 96). A recent study provided cues that GnIH is involved in pubertal disorder induced by abnormalities in the thyroid. The interaction between HPT and HPG axes was reported to mediate via GnIH in abnormal puberty in mice (66), providing molecular targets for the treatment of pubertal disorder resulting from thyroid dysfunction (97). 

**Table 1:** Innervation of RFamide-related peptide (RFRP) neurons in the hypothalamus in different mammalian species

Species	Neuron localization	Neuron projection	Target neurons	References
Human	Dorsomedial region	Preoptic area Median eminence of pituitary	Luteinizing hormone	(23)
Monkey	Pituitary All major types of testicular cells	Hypothalamus Median eminence of pituitary	Gonadotropin-releasing hormone	(22)
Pig	Paraventricular nuclei Dorsomedial nuclei	Lateral hypothalamic area	Gonadotropin-releasing hormone Leydig cells	(17, 18)
Cow	Dorsomedial hypothalamus Paraventricular nuclei	Median eminence of pituitary Preoptic area	Gonadotropin-releasing hormone	(21)
Sheep	Anterior hypothalamus	Mediobasal hypothalamus Paraventricular nuclei Dorsomedial hypothalamus Median eminence of pituitary	Luteinizing hormone	(19, 69)
Goat	Hypothalamus	Median eminence of pituitary	Gonadotropin-releasing hormone	(20)
Rat	Dorsomedial hypothalamic nucleus Arcuate nucleus	Lateral ventricle Paraventricular nucleus Dorsomedial nucleus Arcuate nucleus	Kisspeptin Neuropeptide Y Gonadotropin releasing hormone	(11, 12)
Mouse	Dorsomedial hypothalamic nucleus	Arcuate nucleus Anteroventral-periventricular hypothalamic nuclei	Kisspeptin Gonadotropin releasing hormone Proopiomelanocortin Neuropeptide Y	(13)
Hamster	Dorsomedial hypothalamic nucleus Suprachiasmatic nucleus	Limbic brain regions Septum Striatum Amygdala Hypothalamus Anteroventral–periventricular nucleus Median eminence of pituitary Median preoptic nucleus	Luteinizing hormone Gonadotropin releasing hormone Vasoactive intestinal peptide Anteroventral periventricular nucleus	(5, 10, 15, 16, 61)
Jeroba	Dorso/ventromedialVentro (VMH)-medial Hypothalamus	Arcuate nucleus Periventricular nucleus	Pro-opiomelanocortin Neuropeptide Y Somatostatin Gonadotropin releasing hormone	(14)

**Table 2:** Studies confirmed the inhibitory role of the RFamide-related peptide (RFRP) on the hypothalamus-pituitary-gonad axis during ovarian life-span in different mammalian species

Species	Ovarian lifespan	References
Human	Early puberty	(27)
	Menopause	(23)
Monkey	Prepuberty	(22)
Pig	Estrous cycle	(54, 56, 65)
Cow	Estrous cycle	(21)
Sheep	Estrous cycle	(69)
	Milking	(19)
Goat	Estrous cycle	(20)
Rat	Prepuberty	(11)
	Estrous cycle	(57, 59, 92)
	Pregnancy	(83)
	Milking	(90)
Mouse	Puberty	(26, 39)
	Estrous cycle	(71)
	Pregnancy	(48)
Hamster	Estrous cycle	(5, 60, 61, 74)
Jeroba	Estrous cycle	(14)

*Role of RFRP in infertility*

High psychological anxiety and stress lead to long-lasting impaired fertility, including delayed pregnancy success, and reduced libido to complete reproductive axis suppression particularly in hypothalamic amenorrhea (98). The stress-induced RFRP-3 increase induces long-term suppression of reproductive periods (99). Along this line, there is some evidence that RFRPs could respond differently to any particular stressor, regardless of whether it is chronic or acute (100).

*Role of RFRP in polycystic ovary syndrome*

Polycystic ovary syndrome (PCOS) is a series of symptoms resulting from the increased androgens in women after puberty (101). Many useful clinical manifestations of PCOS are connected with reproductive, endocrine, psychological, and metabolic disorders. Hyperandrogenism and ovulatory dysfunction due to polycystic ovaries are the most prominent features of PCOS (96). Dysregulation of RFRP-3 secretion in the PCOS has been shown (63, 102). A rodent model of PCOS through neonatal chronic testosterone administration is used, causing vaginal opening onset to occur earlier in puberty. Interestingly, the mRNA expression of hypothalamic *RFRP* in testosterone-treated rats is decreased; however, this change stayed unchanged in their serum LH levels. The discovery indicates that sexual maturation in PCOS rats can be created through a peripheral mechanism instead of a central one (63).

The effects of reproductive function and sexual maturation are heavily implicated in the neonatal and/or prepubertal androgen milieu (103). The levels of serum LH and hypothalamic *Kiss1* mRNA expression are decreased and their uterine weight increased in chronic dehydroepiandrosterone (DHEA)-treated rats, suggesting that RFRP-3 might directly affect the regulation of DHEA and/or estrogen in reproductive phenotypes (104). Furthermore, RFRP-3 agonists or LH-secretion related inhibitors may pharmacologically contribute to improving the treatments of steroid-dependent diseases, including endometriosis, benign prostatic hyperplasia, and precocious puberty (29, 105). To specifically address the reciprocal relationship in menstrual cyclicity and levels of plasma kisspeptin, plasma kisspeptin levels of eight female volunteers have been measured (106). Compared with males, the plasma kisspeptin levels are evidently higher in males. However, no significant relationship is found between sex hormones and plasma kisspeptin levels. As suggested by these data, their work reveals that kisspeptin may act as a driver of the beginning of menstruation and advance of menstruation covered for weakened functions of the ovary. Expanding on this research, recordings based on ovarian diseases reveal correlation with RFRP and its receptor. 

RF313, a new orally active neuropeptide FF (NPFF) receptor antagonist, is considered to hinder hyperalgesia development induced by fentanyl in rats. An emerging theme is that RF313 has been proven to serve as a pharmacological tool to research the roles of NPFF1R and RFRPs in regulating reproduction (107). These results provide plausible evidence that RFRP-3 may have the potential for treating hormone-dependent diseases, reproductive diseases and hypothalamus-related ailments, including benign prostatic hyperplasia, uterine fibroids, endometriosis, breast cancers, and precocious puberty; exploring the methods by which the ovarian and reproductive diseases or syndromes in the RFRP system and its modulatory mechanisms represent a current novel wave. Recent studies using pharmacological manipulations unraveled that gonadal RFRP reacts to cues from influences gonadal steroids release and peripheral hormones (108-110). Therefore, there is a crucial need to recognize that involvement of RFRP in female fertility and the RFRP system in reproductive tissues is a potential target for contraceptive or reproductive therapies.

**Conclusions and future outlooks**

Reproduction is regulated via a complex network of stimulatory factors of peripheral and central origins that integrate at the HPG axis. We have witnessed tremendous advances in identifying crucial neuropeptides, RFRPs, involved in coding and processing the mammalian reproductive process. In this review article, the impacts of RFRP on biochemistry and molecular physiology, different periods during mammalian ovarian development in a variety of species, its present application, and therapeutic potential in ovarian diseases were discussed. Further studies are required for exploring whether modulation of RFRP-1 is confined to endocrines without coding the reproductive process, or whether reciprocal or overlapping projections existing between RFRP-3 and RFRP-1 regularize the ovarian lifespan of mammals, or whether RPRP plays an essential role in prenatal and early post-natal ovarian development, or how the neural and gonadal GnIH systems respond to hormones and stressors in different species. Moreover, the physiological mechanisms of the RFRP system in treating mammalian ovarian diseases and reproductive diseases is a brand new exciting frontier awaiting further investigation. 

## Conflicts of Interest

The authors declare that there are no conflicts of interest. 
